# Supplementary material for: The interactome of intact mitochondria by cross-linking mass spectrometry provides evidence for coexisting respiratory supercomplexes
Source: Mol Cell Proteomics. 2017 Dec 8;17(2):216–32. doi: 10.1074/mcp.RA117.000470 (PMC5795388; doi:10.1074/mcp.RA117.000470)
Supplement: Supplemental Data [file supp_RA117.000470_133922_0_supp_23978_fzffwf.zip › spectra_annotation/mito_DR_spectra_annotation/188-1-6-1-10-1.pdf]

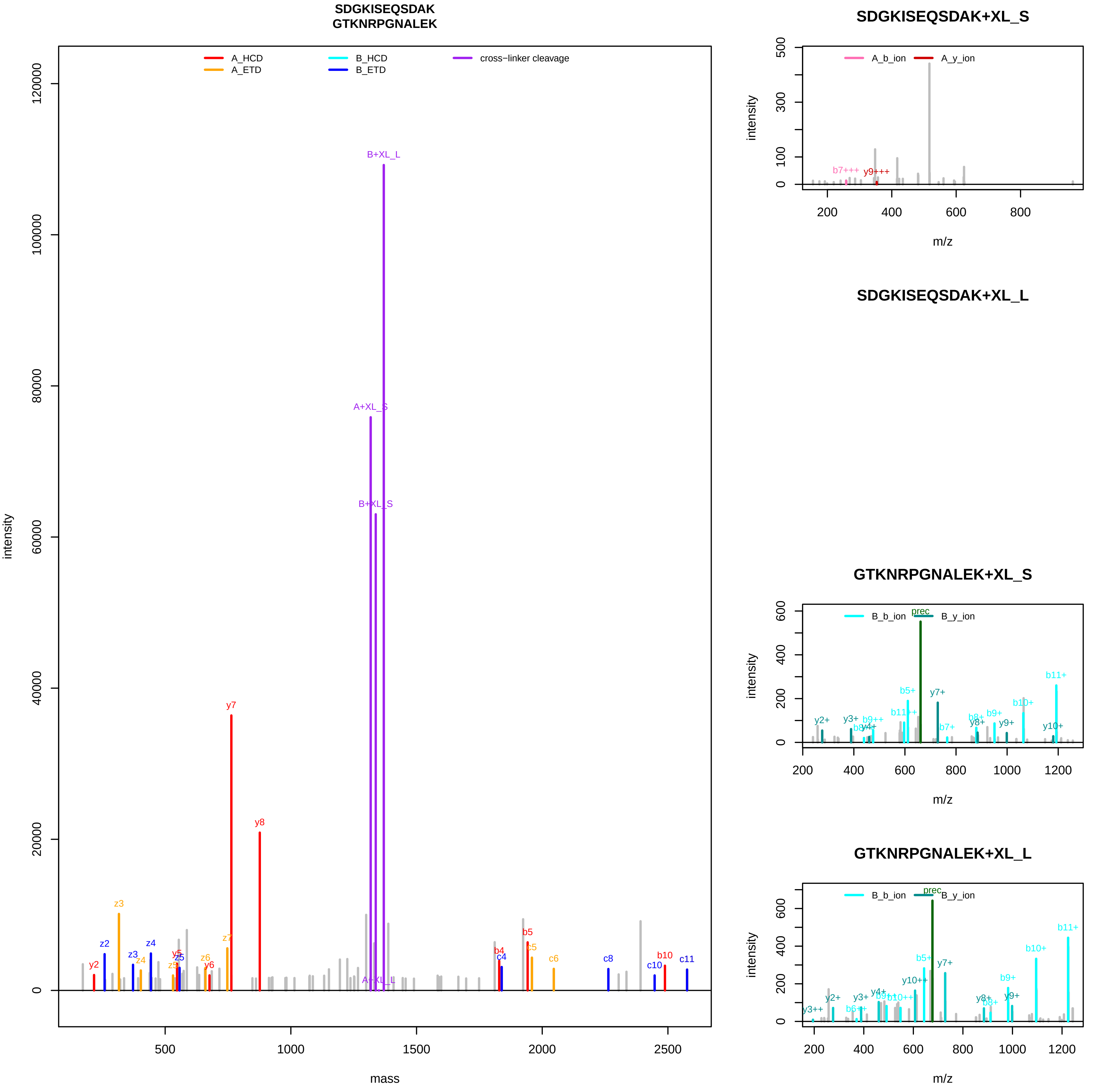

SDGKISEQSDAK
GTKNRPGNALEK
A_HCD
A_ETD
B_HCD
B_ETD
cross−linker cleavage
B+XL_L
A+XL_S
B+XL_S
A+XL_L
y2 z2 z3 z3 z4 z4 z5 y5 z5 z6 y6 z7 y7 y8 b4 c4 b5 c5 c6 c8 c10 b10 c11
intensity
mass
0
20000
40000
60000
80000
100000
120000
500
1000
1500
2000
2500
SDGKISEQSDAK+XL_S
A_b_ion
A_y_ion
b7+++
y9+++
m/z
SDGKISEQSDAK+XL_L
GTKNRPGNALEK+XL_S
B_b_ion
B_y_ion
prec
m/z
GTKNRPGNALEK+XL_L
B_b_ion
B_y_ion
prec
m/z
